# Comparative electrophysiological study of neuroactive steroid-induced hypnosis in mice: sex and drug-specific differences

**DOI:** 10.3389/ebm.2025.10550

**Published:** 2025-06-10

**Authors:** Abigail Martin, Ian Coulter, Reginald Cox, Douglas F. Covey, Slobodan M. Todorovic, Tamara Timic Stamenic

**Affiliations:** ^1^ Department of Anesthesiology, University of Colorado Anschutz Medical Campus, Aurora, CO, United States; ^2^ Department of Developmental Biology, Washington University School of Medicine, Saint Louis, MO, United States; ^3^ Taylor Family Institute for Innovative Psychiatric Research, Washington University School of Medicine, Saint Louis, MO, United States; ^4^ Neuroscience and Pharmacology Graduate Program, University of Colorado Anschutz Medical Campus, Aurora, CO, United States

**Keywords:** neuroactive steroids, hypnosis, electroencephalogram, phase locking value, sex-differences

## Abstract

Since the discovery of their anesthetic effects, some neuroactive steroids have been used as general anesthetics. However, their effects on thalamocortical oscillations and potential sex differences that are associated with their hypnotic/sedative effects are not well studied. Here, we investigated spectral characteristics and sex differences in hypnotic effect of two common neuroactive steroids: Allopregnanolone (AlloP) and its synthetic analog Alphaxalone (Alpx) in wild type mice using behavioral testing (loss of righting reflex - LORR) and *in vivo* electrophysiology. Our data revealed sex-differences in LORR duration with 100 mg/kg intraperitoneally injected AlloP and Alpx confirming that females are more sensitive to neuroactive steroid-induced hypnosis. Spectral analysis, thalamocortical and corticocortical phase synchronization showed notable differences between two neuroactive steroids. AlloP induced a profound reduction in local field potential (LFP) and electroencephalogram (EEG) after LORR with higher LFP/EEG suppression in females during first 60 min after injection. Also, we observed a decrease in thalamocortical synchronization in lower (delta, theta, alpha) and increase in higher low gamma frequency in AlloP group; similar effects were observed in Alpx treated animals with no change in delta thalamocortical phase locking values. Synchronization between right and left cortex was reduced in all frequencies except low gamma in AlloP-treated group. Similarly, Alpx induced reduction in corticocortical synchronization for theta, alpha and beta frequencies. We conclude that AlloP and Alpx have distinct electrophysiological signatures in thalamocortical circuitry that may underly their sedative/hypnotic effects.

## Impact statement

While clinically used general anesthetics are considered relatively safe, there is an ongoing interest in the development of new, potentially safer general anesthetics, especially for specific populations such as children. Neuroactive steroids are utilized as anesthetic agents due to their ability to modulate the activity of γ-aminobutyric acid type A (GABA_A_) receptors in the brain, leading to a sedative and anesthetic effect. Currently, neuroactive steroids are primarily used for the induction and maintenance of general anesthesia in veterinary medicine (Alphaxalone), but there is ongoing interest in exploring their potential for human use as well. Here, we are comparing the spectral characteristics and sex differences in the hypnotic effect of two neuroactive steroids, Allopregnanolone and its synthetic analogue, Alphaxalone. Our data demonstrate that it is important to consider the use of neuroactive steroids for clinical anesthesia, particularly for their potential to be safer than traditional anesthetics.

## Introduction

Neuroactive steroids are endogenous or exogenous steroids with direct effects on neuronal excitability. It has been shown that the spatial arrangement of the hydroxyl group at the C3 position of the steroid skeleton dramatically affects the biological properties of the neuroactive steroids [1, 2]. Previous reports demonstrated that 3α-hydroxy neuroactive steroids, like endogenous Allopregnanolone (AlloP, 3α-hydroxy-5α-pregnan-20-one) or its synthetic analog Alphaxalone (Alpx, 3α-hydroxy-5α-pregnane-11,20-dione), act like positive allosteric modulators of γ-aminobutyric acid type A (GABA_A_) receptors in nanomolar concentrations [3–6]. This effect on γ-aminobutyric acid (GABA) currents is consistent with known behavioral effects of neuroactive steroids including anxiolysis, analgesia, anticonvulsant activity, sedation, hypnosis and anesthesia [7].

Furthermore, neuroactive steroids have been used in human and veterinary clinics as potentially safer anesthetic agents [8]. In comparison to traditional general anesthetics, many neuroactive steroids have favorable clinical properties: minimal cardiorespiratory depression, rapid onset and recovery, less neurotoxicity in young populations [2, 8–10]. It has been shown that AlloP is a potent hypnotic drug not just in wild-type (WT) animals but also in animals that lack progesterone receptors, suggesting that progesterone receptors are not involved in the hypnotic response to AlloP [11, 12]. Similarly, a synthetic drug, Alpx, has potent hypnotic/anesthetic and sedative properties [2, 8]. Although it was abandoned for clinical use due to unwanted side effects caused by its vehicle, there has been recent interest in the therapeutic value of Alpx: a new aqueous formulation (Phaxan^®^) has been developed for use as an intravenous sedative and anesthetic in humans [9, 13]. While the sex-differences in various behavioral effects of neuroactive steroids are well documented in the literature, their neurophysiological signature is poorly understood [14].

Central medial nucleus of thalamus (CMT), as a part of intralaminar thalamic complex is implicated in arousal and is a key hub through which general anesthesia and sleep are initiated [15]. Here, we investigated sex-differences and spectral characteristics in the hypnotic effect of AlloP and Alpx in WT mice using behavioral testing (loss of righting reflex - LORR) and *in vivo* electrophysiology by recording local field potential (LFP) from CMT and electroencephalogram (EEG) from barrel cortex. Since there is an ongoing interest in developing new hypnotics and general anesthetics, we hope that our study may rekindle interest in clinical use of these agents as safer alternative to other common sedatives and anesthetics.

## Materials and methods

All experimental procedures with mice were performed according to a protocol approved by the Institutional Animal Care and Use Committee of the University of Colorado Anschutz Medical Campus, Aurora, CO, USA. Treatments of animals adhered to guidelines set forth in the NIH Guide for the Care and Use of Laboratory Animals. All efforts were made to minimize animal suffering and to use only the necessary number of animals to produce reliable scientific data. The authors confirm that the study complies with the ARRIVE guidelines.

Male and female adult C57BL/6J WT 3–4 months old mice (Jackson Laboratory, Bar Harbor, ME, USA) were used for behavioral and *in vivo* electrophysiology studies. All mice were maintained on a 14/10h light-dark cycle with food and water *ad libitum*. All tests were done in a blinded fashion. The estrus cycle of the female animals was not monitored during the experiments and can be a limitation of the study.

### Neuroactive steroid preparation

**FIGURE 1:**
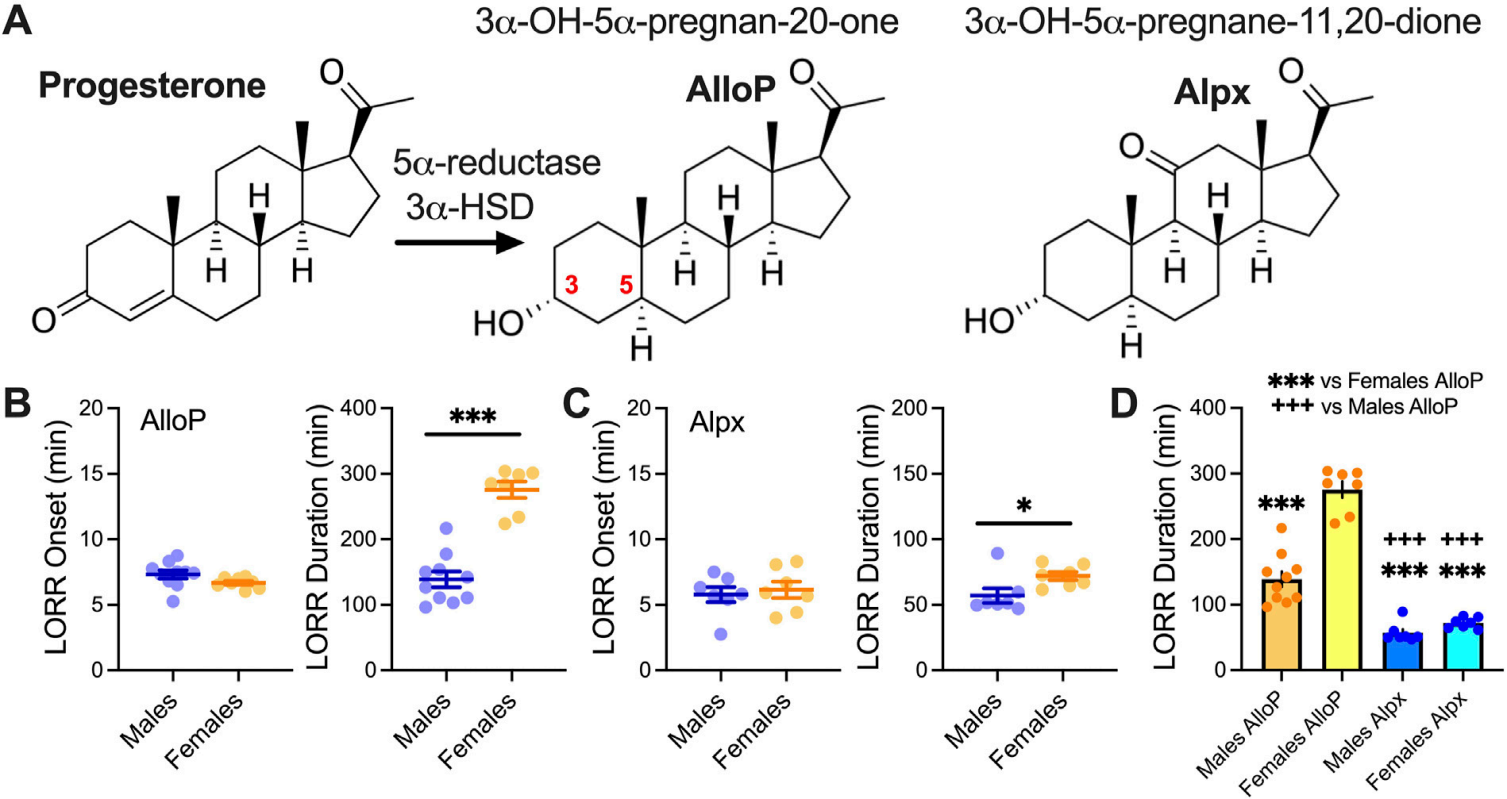
LORR with 100 mg/kg AlloP and Alpx. **(A)** Schematic presentation of AlloP synthesis from progesterone (3α-hydroxysteroid dehydrogenase - 3α-HSD) and AlloP synthetic analog Alpx. **(B)** Left LORR onset with 100 mg/kg AlloP (10 male and 7 female animals); **(B)** Right LORR duration with 100 mg/kg AlloP; unpaired two-tailed t-test t_(15)_ = 7.74, p < 0.001. **(C)** Left LORR onset with 100 mg/kg Alpx (7 mice per group); **(C)** Right LORR duration with 100 mg/kg Alpx; unpaired two-tailed t-test t_(12)_ = 2.36, p = 0.036. **(D)** Differences between AlloP and Alpx in LORR duration, one-way ANOVA F(3,27) = 89.64, p < 0.001, Tukey’s *post hoc* presented on Figure. Blue-males, orange-females *p < 0.05, ***p < 0.001.

AlloP and Alpx (Figure 1A) were synthetized and obtained from Dr. Covey (Saint Louis, MO, USA) and were dissolved in 25% (2-Hydroxypropyl)-β-cyclodextrin (Santa Cruz Biotechnology Inc, Dallas, TX, USA) in H_2_O to yield the desired concentration for intraperitoneal (i.p.) injection. The solutions were prepared on the same day as they were injected into the mice. Mice were weighed and injected with the appropriate volume of neuroactive steroids to achieve desired dose (100 mg/kg). Our preliminary experiments showed that with this dose all injected animals exhibited loss of righting reflex (LORR).

### Assessment of hypnotic behavior in mice

One desirable endpoint of general anesthesia is the state of unconsciousness, also known as hypnosis [16]. A widely used behavioral surrogate for hypnosis in rodents is the loss of righting reflex (LORR), or the point at which the animal no longer responds to their innate instinct to avoid the vulnerability of dorsal recumbency [16]. We measured hypnosis by assessing the LORR. After 30 min of habituation to the testing chamber male and female mice received i.p. injections of 100 mg/kg AlloP or Alpx and placed on a heating pad in a clear plastic cage. This cage was intermittently gently tilted to roll a given mouse onto its back. Loss of righting reflex was measured as inability to right self within 30 s. To assess duration of the LORR we measured the gain of righting reflex time. Animals were placed on a heating pad to prevent hypothermia and oxygen levels were checked intermittently during LORR experiments using pulse oximetry.

### EEG data acquisition and spectral analysis

Synchronized, time locked video and EEG/LFP signals were recorded using the Pinnacle system (Pinnacle Technology Inc., Lawrence, KS, USA). The EEG and LFP signals were amplified (100×) and digitized at a sampling frequency rate of 2000 Hz (high pass filter 0.5 Hz and low pass filter 500 Hz) and stored on a hard disk for offline analysis. The electrodes (two screw-type cortical [AP: −1 mm, MD: ± 3 mm, DV: 0]) and one depth coated tungsten in CMT [anteroposterior–AP: −1.35 mm, mediolateral–MD: 0 and dorsoventral–DV: −3.6 mm], were implanted under continuous 2.5 vol% isoflurane anesthesia. Banamine^®^ – Merck (i.p. 2.5 mg/kg) was applied right after surgery and every 24 h for 48 h. Seven to 10 days after surgery animals were put in the recording chamber ((H × W × L) 15.2 × 16.5 × 31.1 cm) and EEG/LFP were recorded 30 min before (baseline recordings) and 60 min after i.p. injection of neuroactive steroid. To compare spectra, 5 min of signal in baseline (wake state) and during 100 mg/kg i.p. of neuroactive steroid were analyzed. After completion of experiments, mice were briefly anesthetized with ketamine (100 mg/kg i.p.) and electrolytic lesions were made by passing 5 μA current for 1 s (5 times) to verify placement of the deep electrode. Mice were anesthetized additionally with isoflurane and perfused with ice-cold 0.1M phosphate buffer containing 1% of potassium-ferrocyanide. The brains were extracted, kept in 4% formalin (PFA) for 2 days and sliced (100–150 μm) using a vibrating micro slicer (Laica VT 1200 S). Photos of coronal slices with electrode location conformation were obtained using bright-field Zeiss stereoscope and Zen Blue software.

We excluded thalamic recordings from 4 animals in the analysis; one female animal that did not have good deep (CMT) electrode placement, two female and one male animal did not have good quality of thalamic recording.

### Data analysis

Statistical analysis was performed using one- or two-way repeated measure (RM) ANOVA as well as Student unpaired two-tailed t-test where appropriate. Generally one-way RM ANOVA was used for LORR analysis between neuroactive steroids, two-way RM ANOVA for spectral analysis, and unpaired t-test for sex-differences in LORR. Where interaction between factors after two-way RM ANOVA was significant, Sidak’s multiple comparisons test was used. If one-way ANOVA was significant, Tukey’s comparison test was used. Significance was accepted with *p* values <0.05. Statistical and graphical analysis was performed using GraphPad Prism 8.00 software (GraphPad Software, La Jolla, CA, USA). The EEG frequency spectrum was divided into the following frequency bands: delta (0.5–4 Hz), theta (4–8 Hz), alpha (8–13 Hz), beta (13–30 Hz) and low gamma (30–50 Hz). Power density, total power and relative power spectral analysis were calculated using LabChart 8 software (ADInstruments Inc., Colorado Springs, CO). For additional EEG/LFP analysis we used Brainstorm software package implemented in MATLAB [17, 18]. We calculated thalamocortical and corticocortical phase locking values for functional connectivity using the Hilbert transform based phase synchronization analysis [19]. The phase locking values were used as non-directed functional connectivity metrics to capture interdependence between two signals–thalamocortical (CMT and cortex) and corticocortical (right and left cortex) [20]. All data are presented as mean ± SEM.

### Data availability

Data will be made available from the corresponding author on request.

## Results

### Sex-differences in LORR after 100 mg/kg AlloP and Alpx injections

Figure 1A shows chemical structure of neuroactive steroids and a biosynthesis of the AlloP from progesterone; AlloP is derived from progesterone by reduction at the 5- and 3-positions of the steroid A-ring via 5α-reductase and 3α-hydroxysteroid dehydrogenase [7]. We examined the effect of 100 mg/kg AlloP and Alpx on LORR in male and female mice (Figures 1B,C). All tested animals lost righting reflex after drug injection. While the onset of LORR was not changed, both neuroactive steroids showed a sex-dependent effect in LORR duration. Specifically, female mice had longer times for LORR duration than males (Figures 1B,C). Interestingly, both male and female animals injected with the 100 mg/kg AlloP had longer LORR durations in comparison to mice injected with Alpx. Furthermore, sex differences were more notable in AlloP group where LORR in females was about 2-fold longer than in males (Figure 1D). Onset of LORR was not different between different sexes for both neuroactive steroids.

### Changes in total thalamic and cortical oscillatory power under AlloP and Alpx over time

**FIGURE 2:**
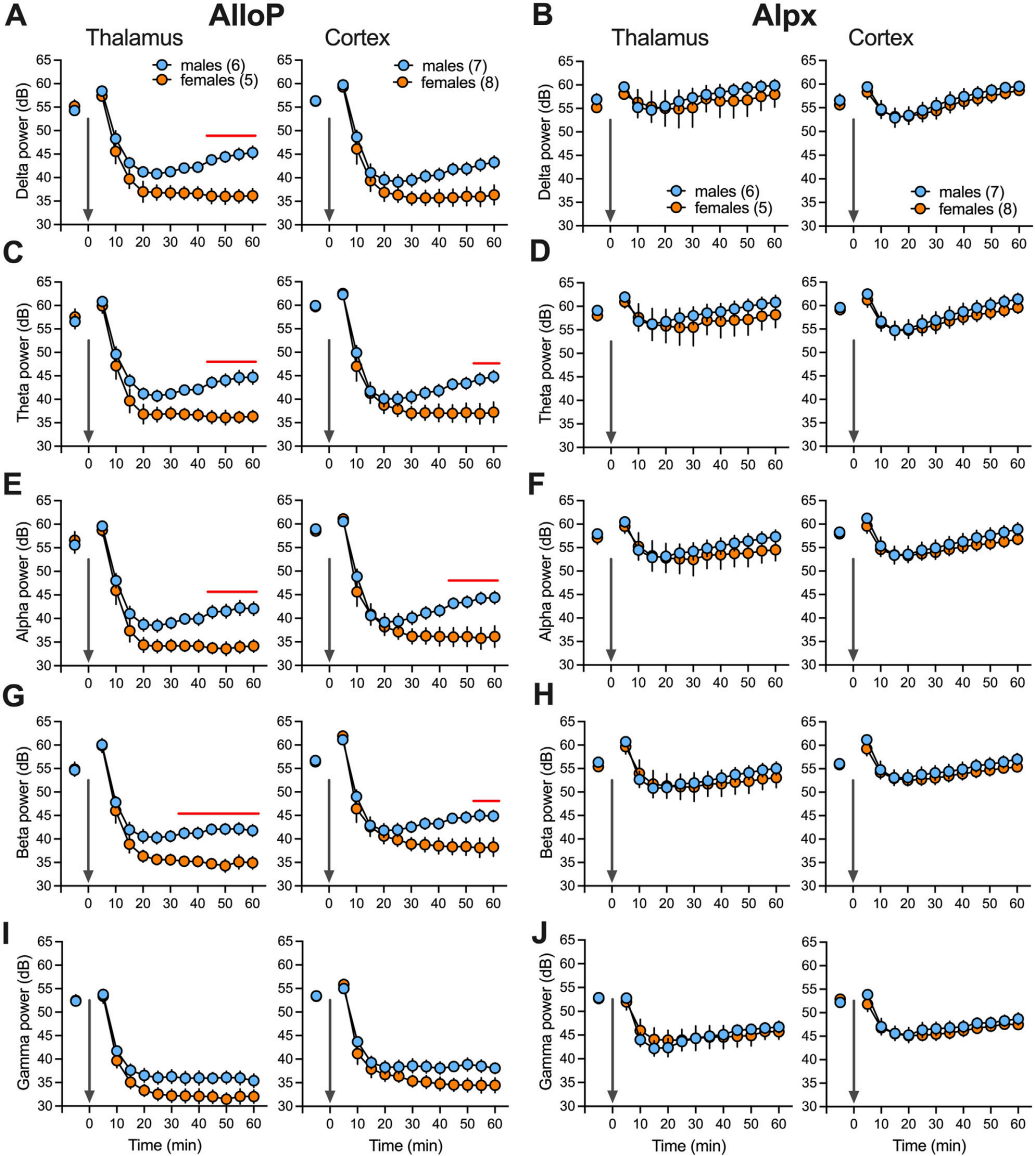
Total power changes after neuroactive steroids over time (60 min). Total (dB) thalamic (left) and cortical (right) delta **(A)**, theta **(C)**, alpha **(E)**, beta **(G)** and low gamma **(I)** power during wake and after 100 mg/kg AlloP. Statistical analysis: two way RM ANOVA for thalamic delta power: interaction F_(12,108)_ = 6.55, p < 0.001, time F_(12,108)_ = 112, p < 0.001, sex F_(1,9)_ = 10.31, p = 0.011, Sidak’s *post hoc* presented on figure); two way RM ANOVA for thalamic theta power: interaction F_(12,108)_ = 4.56, p < 0.001, time F_(12,108)_ = 117.2, p < 0.001, sex F_(1,9)_ = 8.35, p = 0.018, Sidak’s *post hoc* presented on figure; two way RM ANOVA for cortical theta power: interaction F_(12,156)_ = 4.05, p < 0.001, time F_(12,156)_ = 142.6, p < 0.001, sex F_(1,13)_ = 3.24, p = 0.095, Sidak’s *post hoc* presented on figure; two way RM ANOVA for thalamic alpha power: interaction F_(12,108)_ = 4.48, p < 0.001, time F_(12,108)_ = 129.9, p < 0.001, sex F_(1,9)_ = 8.18, p = 0.019, Sidak’s *post hoc* presented on figure; two way RM ANOVA for cortical alpha power: interaction F_(12,156)_ = 5.64, p < 0.001, time F_(12,156)_ = 128.7, p < 0.001, sex F_(1,13)_ = 3.72, p = 0.076, Sidak’s *post hoc* presented on figure; two way RM ANOVA for thalamic beta power: interaction F_(12,108)_ = 4.7, p < 0.001, time F_(12,108)_ = 139.2, p < 0.001, sex F_(1,9)_ = 9.53, p = 0.013, Sidak’s *post hoc* presented on figure; two way RM ANOVA for cortical beta power: interaction F_(12,156)_ = 4.66, p < 0.001, time F_(12,156)_ = 121.4, p < 0.001, sex F_(1,13)_ = 3.47, p = 0.085, Sidak’s *post hoc* presented on figure. Total thalamic (left) and cortical (right) delta **(B)**, theta **(D)**, alpha **(F)**, beta **(H)** and low gamma **(J)** power during wake and after 100 mg/kg Alpx. Note that we did not observe statistical difference between male and female animals after Alpx during first 60 min in all analyzed frequencies. Blue-males, orange-females, number of mice per group are presented on figure, red line presents statistically significant *post hoc* test.

To analyze further sex-differences during AlloP- and Alpx-induced hypnosis we recorded LFP from CMT and EEG from right and left barrel cortex. CMT has been previously implicated in the mechanisms of both general anesthesia and sleep initiation [15]. In animals that received 100 mg/kg AlloP, we observed a very strong LFP/EEG suppression of total power (dB) in all analyzed frequencies after LORR during the first 60 min after injection (Figures 2A,C,E,G,I). By contrast, animals that were injected with 100 mg/kg Alpx showed very little change in LFP/EEG signals after LORR in comparison to the wakeful state (Figures 2B,D,F,H,J). Consistent with its longer hypnotic effect, suppression under AlloP was more profound in females in thalamic delta (Figure 2A left), thalamic and cortical theta, alpha and beta power (Figures 2C,E,G, respectively). On the contrary, we did not observe sex differences in LFP and EEG recordings after Alpx injection.

### Spectral signatures and spectrograms 30 min after AlloP and Alpx injection

**FIGURE 3:**
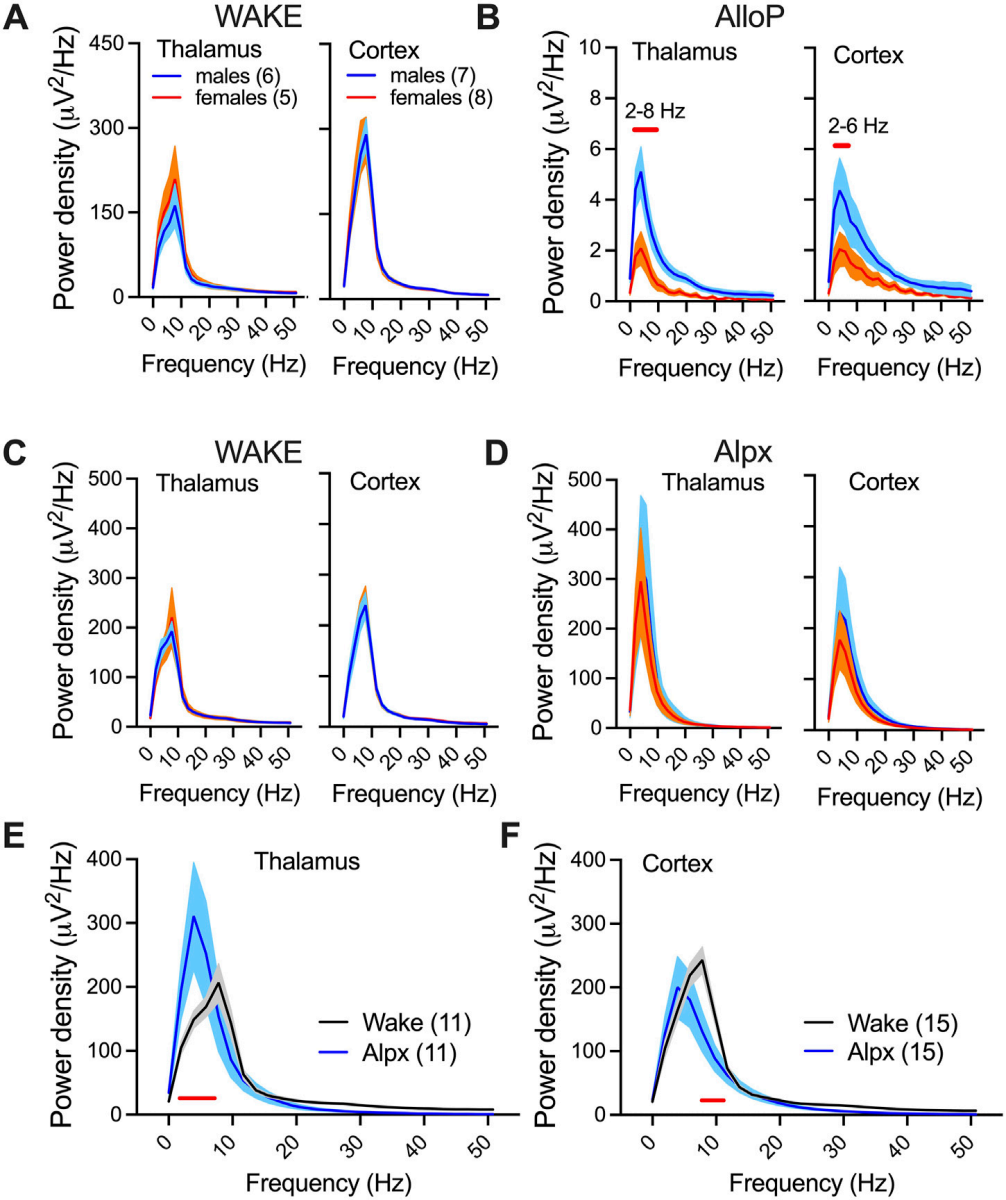
Spectral changes and sex-differences under AlloP and Alpx. Thalamic (left) and cortical (right) power densities during wake state **(A)** and 25–30 min after 100 mg/kg AlloP injection **(B)**. Female mice had reduction in 2–10 Hz thalamic and 2–6 Hz cortical power densities in comparison to male mice under AlloP; two way RM ANOVA for thalamic power density: interaction F_(26,234)_ = 4.75, p < 0.001, frequency F_(26,234)_ = 27.22, p < 0.001, sex F_(1,9)_ = 8.7, p = 0.016, Sidak’s *post hoc* presented on figure; two way RM ANOVA for cortical power density: interaction F_(26,338)_ = 1.74, p = 0.016, frequency F_(26,338)_ = 13.72, p < 0.001, sex F_(1,13)_ = 4.53, p = 0.053, Sidak’s *post hoc* presented on figure. **E**, Thalamic (left) and cortical (right) power densities during wake state **(C)** and 25–30 min after 100 mg/kg Alpx injection **(D)**. We did not observe sex differences in power densities after Alpx and analyzed females and males together **(E,F)**. Alpx-induced changes in power density in thalamus **(E)**; two way RM ANOVA: interaction F_(26,234)_ = 3.37, p < 0.001, frequency F_(26,234)_ = 23.95, p < 0.001, Alpx F_(1,9)_ = 0.05, p = 0.83, Sidak’s *post hoc* significant 2–6 Hz; and cortex **(F)**; two way RM ANOVA: interaction F_(26,338)_ = 2.72, p < 0.001, frequency F_(26,234)_ = 75.70, p < 0.001, Alpx F_(1,13)_ = 1.03, p = 0.329, Sidak’s *post hoc* significant 8–10 Hz. Blue-males, orange-females, number of mice per group are presented on figure; red line presents statistically significant *post hoc* test.

We chose 25–30 min after injection to further investigate neuroactive steroid-induced spectral changes (Figure 3). During wakeful periods before AlloP and Alpx injection we did not observe sex-differences in either thalamic or cortical power densities (Figures 3A,C). However, AlloP induced a profound suppression of power densities in comparison to wake state in both thalamus and cortex. Power densities in both the thalamus (2–8 Hz frequency range) and cortex (2–6 Hz frequency range, Figure 3B) were about 2-fold higher in male mice after AlloP injection. Moreover, there was a shift in maximal (peak) power density towards slower oscillations, from dominant wake theta frequency (8Hz, Figure 3A) to dominant delta oscillations during AlloP exposure (4Hz, Figure 3B). The same shift in maximal power density peak was observed with Alpx (Figures 3E,F), except instead of suppression in power density, increase in slower frequencies (2–6 Hz) was observed in thalamus in comparison to wake state (Figure 3E).

### Difference in relative power and functional connectivity between two neuroactive steroids

**FIGURE 4:**
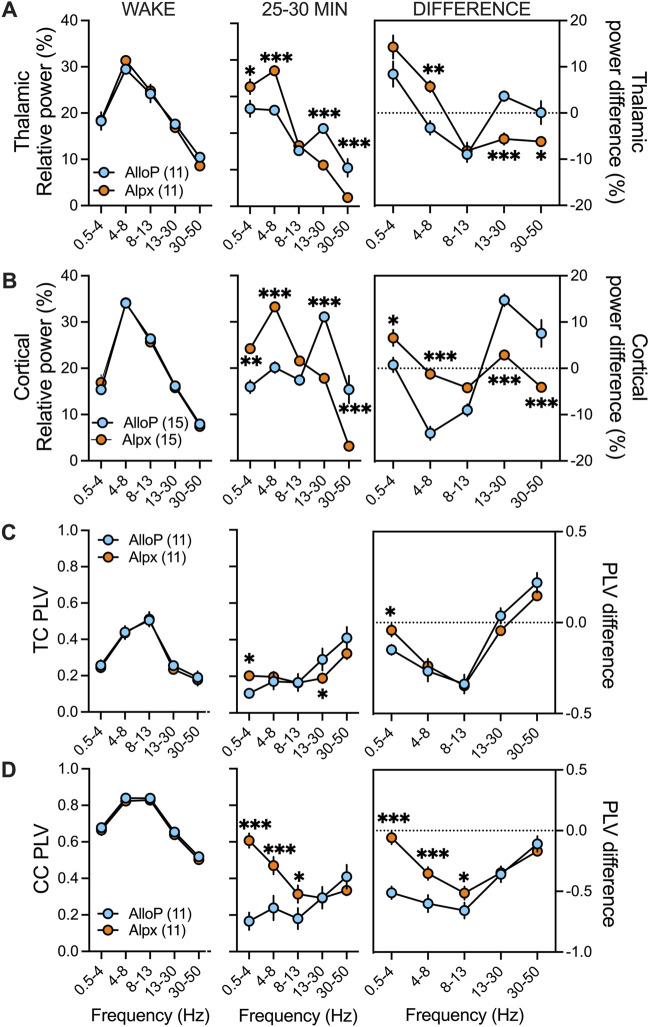
Differences in spectral characteristics between AlloP and Alpx. Since there was no statistical significance in thalamocortical (TC) and corticocortical (CC) phase locking values (PLVs) and relative power (%) between sexes during wake periods and 25–30 min after neuroactive steroid injection both female and male data are combined together for this figure. **(A)** Thalamic relative power during wake state (left), after AlloP and Alpx injection (middle) and change in relative power from wake state (right). We did not observed differences during wake periods. Animals injected with Alpx had higher delta and theta but lower beta and low gamma relative powers in comparison to Alpx group; two-way RM ANOVA: interaction F_(4,40)_ = 22.17, p < 0.001, frequency F_(4,40)_ = 56.24, p < 0.001, neuroactive steroid F_(1,10)_ = 0.56, p = 0.471, Sidak’s *post hoc* presented on figure. Change in relative power from wake state showed increase in Alpx theta and decrease in beta and low gamma thalamic relative power that was not seen in Allop treated animals; two-way RM ANOVA: interaction F_(4,40)_ = 11.79, p < 0.001, frequency F_(4,40)_ = 19.02, p < 0.001, neuroactive steroid F_(1,10)_ = 0.52, p = 0.486, Sidak’s *post hoc* presented on figure **(B)** Cortical relative power during wake state (left), after AlloP and Alpx injection (middle) and change in relative power from wake state (right). We did not observed differences during wake periods. Animals injected with Alpx had higher delta and theta but lower beta and low gamma relative powers in comparison to Alpx group; two-way RM ANOVA: interaction F_(4,56)_ = 33.92, p < 0.001, frequency F_(4,56)_ = 44.49, p < 0.001, neuroactive steroid F_(1,14)_ = 4.1, p = 0.062, Sidak’s *post hoc* presented on figure. Interestingly, AlloP treated animals showed increase in beta and low gamma relative powers from wake state and decease in beta relative power while animals in Alpx group had increase in delta cortical relative power that was not seen with AlloP; two-way RM ANOVA: interaction F_(4,108)_ = 21.37, p < 0.001, frequency F_(4,108)_ = 33.63, p < 0.001, neuroactive steroid F_(1,27)_ = 1.54, p = 0.226, Sidak’s *post hoc* presented on figure. **(C)** TC PLVs during wake (left), after AlloP or Alpx (middle) and change in PLV from wake state (right). We did not observe PLVs differences during wake state. There was increase in delta and decrease in beta TC PLVs in Alpx group in comparison to AlloP; two-way RM ANOVA: interaction F_(4,40)_ = 6.298, p = 0.001, frequency F_(4,40)_ = 21.19, p < 0.001, neuroactive steroid F_(1,10)_ = 0.13, p = 0.725, Sidak’s *post hoc* presented on figure. Analysis of PLV difference showed decrease in delta TC PLVs in AlloP but not in Alpx group; two-way RM ANOVA: interaction F_(4,40)_ = 4.78, p = 0.003, frequency F_(4,40)_ = 59.60, p < 0.001, neuroactive steroid F_(1,10)_ = 0.03, p = 0.876, Sidak’s *post hoc* presented on figure **(D)** CC PLVs during wake (left), after AlloP or Alpx (middle) and change in PLV from wake state (right). We did not observe PLVs differences during wake state. There was increase in delta, theta and alpha CC PLVs in Alpx group in comparison to AlloP; two-way RM ANOVA: interaction F_(4,40)_ = 5.71, p < 0.001, frequency F_(4,40)_ = 5.71, p = 0.001, neuroactive steroid F_(1,10)_ = 7.90, p = 0.019, Sidak’s *post hoc* presented on figure. Similarly, analysis of PLV difference showed more profound decrease in delta, theta and alpha CC Plvs after AlloP than after Alpx injection; two-way RM ANOVA: interaction F_(4,40)_ = 15.98, p < 0.001, frequency F_(4,40)_ = 36.64, p < 0.001, neuroactive steroid F_(1,10)_ = 10.76, p = 0.008, Sidak’s *post hoc* presented on figure. Blue-males, orange-females, number of mice per group is presented on figure, *p < 0.05, **p < 0.01, ***p < 0.001.

We did not detect significant sex-differences in relative power and corticocortical and thalamocortical phase locking values before or under neuroactive steroids, hence we combined the data from male and female mice to perform statistical analysis (Figure 4). We found no significant differences in thalamic and cortical relative powers during baseline (wake state) between the two neuroactive steroids (Figure A,B left). Both AlloP and Alpx increased delta and decreased alpha relative power in thalamus (Figure 4A right). Alpx increased thalamic theta and decreased beta and low gamma relative power (Figure 4A right). In cortex, delta relative power was increased only in the Alpx group but not in the AlloP-treated mice (Figure 4B right). However, AlloP decreased beta, alpha and increased beta and low gamma cortical relative powers (Figure 4B right). Additionally, Alpx-treated group had higher relative powers of slower frequencies (delta and theta) and had lower relative powers of higher frequencies (beta and low gamma) in comparison to AlloP in thalamus (Figure 4A middle) and cortex (Figure 4B middle).

To investigate functional thalamocortical and corticocortical connectivity we analyzed phase locking values before (wake) and after AlloP and Alpx injections. We detected about 3-fold higher corticocortical phase locking values in comparison to thalamocortical phase locking values during wake stage (Figures 3C,D left). Under AlloP there was a decrease in slower frequencies (delta, theta and alpha) and an increase in low gamma thalamocortical phase locking values (Figure 4C right). Similar results were observed with Alpx except for no change in delta thalamocortical phase locking values (Figure 4C, right). Additionally, there was a decrease in delta, theta, alpha and beta corticocortical phase locking values under AlloP (Figure 4D right). Similarly to thalamic data, Alpx did not change delta corticocortical phase locking values but decreased corticocortical synchronization for beta, alpha and beta frequency range (Figure 4D right). Alpx-treated mice also had higher delta and lower beta thalamocortical phase locking values (Figure 4C middle), and higher delta, theta and alpha corticocortical phase locking values in comparison to AlloP injected animals (Figure 4D middle).

## Discussion

Although the introduction of Alpx and related 3α-OH analogues with hypnotic/anesthetic properties occurred in the 1970s, there is an increased interest recently in the development of new neuroactive steroids as therapeutic agents. For example, AlloP was formulated and FDA-approved as a medicine for postpartum depression (Brexanolone^®^), and a 3β-methylated analogue of AlloP (Ganaxolone^®^) was FDA-approved for treatment of seizure associated with the rare disease cyclin-dependent kinase-like 5 deficiencies [10]. Furthermore, a new aqueous formulation of Alpx (Phaxan^®^) has been developed for use as an intravenous sedative and anesthetic in humans [9, 13].

As expected, we found that both AlloP and Alpx can reliably produce LORR in male and female mice. We further found that there are notable sex-differences in the effects of both neuroactive steroids in tested behavior. Specifically, the female animals are more sensitive to the hypnotic effect. Previous studies reported sex specific effect with both endogenous neuroactive steroids and synthetic analogues [21, 22]. The longer duration of LORR in females was previously described with Alpx in both mice and rats [22–24]. It was postulated that the interactions of neuroactive steroids with synaptic membranes may be more sex-specific [22, 23] and can potentially explain why the female mice in our study had a longer duration of hypnosis with the same dose of AlloP and Alpx. The authors found that the sex differences with Alpx is age-dependent, can be abolished by administering estrogen to the males, does not depend on sexual differentiation of the brain, and cannot be attributed to a sex difference in the metabolic clearance rate of neuroactive steroids [22].

Similarly, it was reported that neuroactive steroids such as AlloP exhibit sex differences in their anticonvulsant activity indicating reduced potency in the males relative to females [25]. This effect can be likely due to a greater abundance of extra-synaptic δ subunit of GABA_A_ receptors that mediates neuroactive steroid-sensitive, tonic GABA currents and seizure protection [26] and can at least partially explain sex specific effect in our experiments.

Interestingly, recent studies demonstrated that the female brain in mice and humans is less responsive to the hypnotic effects of volatile anesthetics largely due to acute effects of sex hormones [27]. Despite clear behavioral sex differences in anesthetic sensitivity, sex-differences were not visible in clinically used EEG but were detected in subcortical recordings [27]. This hidden resistance to volatile anesthetics may explain the higher incidence of awareness under anesthesia in females and requires investigation of sex-differences using both EEG (cortical) and LFP recordings [27].

Neuronal inhibition in the brain is predominantly provided by activation of GABA_A_ receptors - heteropentameric receptors formed from an array of subunits (α1-6, β1-3, γ1-3, δ, ε, π, θ and ρ1-3) [28, 29]. Synaptic receptors typically comprise α1-3, β and γ subunits, while extra-synaptic receptors contain α4/6, β and δ subunits [30]. It has been shown that AlloP has higher selectivity for tonic δ-containing GABA_A_ receptors, and that by being more selective to extra-synaptic GABA_A_ receptors, AlloP plays an essential neurophysiological role in the fine-tuning of neuronal inhibition mediated by GABA_A_ receptors [31, 32]. However, when injected in high doses, as in our case (100 mg/kg, i.p.), AlloP produced drastic suppression of total power in both male and female mice after LORR probably by acting on both synaptic and extra-synaptic GABA_A_ receptors. The clinically relevant dose range for AlloP in rodents varies depending on the desired effect and route of administration, and for anesthetic effect is higher (15–20 mg/kg i.v) than for anxiolytic/sedative effect in animals [28]. Additionally, the typical dose range for Alpx with hypnotic effect is between 50 and 60 mg/kg i.p. in females but higher doses are needed for male rodents [23]. To compare hypnotic effect of AlloP and Alpx, based on our preliminary dose-response experiments, we used the lowest dose of neuroactive steroids that induces LORR in all tested female and male mice.

Since the introduction of EEG, neural oscillations have been systematically categorized across behavioral states into canonical frequency bands: delta, theta, alpha, beta, gamma [33]. Both natural sleep and general anesthesia are associated with synchronized, high-amplitude slow oscillations, whereas conscious wakefulness is dominated by faster, desynchronized activity patterns [34]. Low doses of sedative-hypnotic agents, such as positive allosteric modulators of GABA_A_ receptors (e.g., propofol, barbiturates, etomidate), can paradoxically induce cortical excitation, typically reflected as enhanced β-band activity [35]. As anesthetic depth increases, EEG signatures progress through characteristic stages: Phase 1 (light sedation) is marked by reduced beta and elevated alpha power; Phase 2 (intermediate depth) displays prominent alpha and delta oscillations, closely resembling non-rapid eye movement slow-wave sleep; Phase 3 exhibits burst-suppression patterns; and Phase 4 is characterized by a near-isoelectric EEG, indicative of profound cortical silencing [35]. Although full anesthesia can be assessed by investigating withdrawal reflex to a painful stimulus, like tail or toe clamping and pinching, here we investigated just the hypnotic effect and were unable to assess if AlloP or Alpx induce surgical levels of anesthesia in high dose. However, strong EEG/LFP suppression that we observed with AlloP resembles burst suppression seen with potent volatile general anesthetics [36]. Similarly to sex-differences observed in behavioral test, the EEG/LFP suppression seen with AlloP was more profound in female animals in comparison to males. On the contrary, with the same dose of Alpx we did not observe LFP/EEG suppression, confirming previous results that Alpx alone can induce hypnosis or light anesthesia but even in higher doses probably cannot induce deeper surgical levels of general anesthesia [23, 24]. The observed changes between AlloP and Alpx-induced hypnosis could be at least partially explained by the differences in modulation of synaptic α_1_β_2_γ_2_ GABA_A_ receptors; AlloP is a more potent positive allosteric modulator of these receptors than Alpx (ED_50_ for α_1_ containing GABA_A_ receptors for AlloP 0.18 ± 0.002 μM (mean ± SEM) and for Alpx 2.2 ± 0.04 μM) [37]. The same rationale can explain longer LORR durations induced by AlloP in comparison to Alpx in both female and male mice. Somewhat surprisingly, sex-differences in total EEG/LFP power in our experiments were not seen with the Alpx.

In the cortex and thalamus, power density analysis showed a shift in the power density peak from theta during wakefulness to delta frequencies after both neuroactive steroids, accompanied with a drastic suppression of EEG/LFP power density only in the AlloP group. On the contrary, thalamic spectral analysis showed increase in power density in slower frequencies under Alpx in comparison to the wake state. Similarly to other general anesthetics that act as GABA_A_ positive allosteric modulators, analysis of relative power showed increase in thalamic relative delta power in AlloP and Alpx animals while increase in EEG delta relative power was observed just with Alpx [38, 39]. Although both alpha and slow-delta oscillations are dominant EEG signature for propofol-induced hypnosis and hypnosis with other GABA_A_ positive allosteric modulators, with neuroactive steroids we observed a decrease and not an increase in thalamic and cortical alpha relative power [38, 39]. The difference in effect between neuroactive steroids and other GABA_A_ positive allosteric modulators was previously reported and might be related to effects on different GABA_A_ receptor isoforms [2, 10, 40, 41]. Additionally, neuroactive steroids have higher affinity for extra-synaptic GABA_A_ receptors responsible for persistent non-desensitizing inhibitory conductance [42]. Interestingly, we observed a substantial rise in relative cortical beta/low gamma powers after AlloP-induced LORR. A similar rise in low gamma range was seen in rodents after injections of ketamine, an N-methyl-D-aspartate (NMDA) antagonist [43, 44]. By contrast, we found that Alpx decreased thalamic beta and slightly increased cortical beta relative power and reduced low gamma relative power in both the thalamus and cortex. Overall, our data showed that in AlloP-treated mice, relative powers of slower (delta and theta) frequencies are lower but relative powers of higher (beta and low gamma) frequencies are higher than in the Alpx group.

Additionally, we used the Hilbert transform based phase synchronization analysis [19], to investigate the phase coupling during neuroactive steroid-induced hypnosis. Systematic phase synchronization changes were detected after injection of both neuroactive steroids. Generally, we observed reduction in corticocortical phase synchronization in AlloP animals that was more profound in slower than in higher frequency ranges. Similarly, Alpx induced drop in corticocortical synchronization in all except delta frequencies. We observed existence of higher corticocortical phase locking values under Alpx in comparison to AlloP in delta, theta and alpha frequencies. Previous models predicted that anesthesia would induce a decrease in slow wave EEG phase coherence as the cortex transitions away from the wakeful state [45]. In line with these findings, some experiments have confirmed that phase coherence during induction with propofol produces a significant drop in slow-wave bands synchronization between frontal, occipital, and frontal-occipital electrode pairs in humans [45, 46]. With AlloP we observed a similar decrease in cortical delta phase locking values. Previous findings reported that traditional general anesthetics can induce a large and localized increase in synchronization in alpha band as well as smaller and widespread synchrony increases in gamma frequency bands [47]. Studies using propofol in humans have reported that alpha band synchronization between cortical sites increases during induction and decreases during recovery [46]. By contrast, with neuroactive steroids we observed a profound decrease in alpha frequency synchronization in both corticocortical and thalamocortical leads in all treatment groups. Interestingly, we found increased synchronization in thalamocortical low gamma oscillation in both AlloP and Alpx treatment groups. This finding further denotes that neuroactive steroids induce very distinctive spectral changes that were not described with other similar GABA-mimetic general anesthetics.

Furthermore, in contrast to commonly used general anesthetics, both AlloP and Alpx activate progesterone receptors, and have neuroprotective effects that is independent of their GABA_A_ actions [48]. Recently, it has been shown that Alpx treated subjects scored better than propofol and sevoflurane anesthetized patients in the cognition tests, and that the higher cognition scores were accompanied by higher serum m-Brain-Derived Neurotrophic Factor levels in the Alpx-anesthetized patients [13]. Having this in mind, we posit that use of neuroactive steroids as general anesthetics may have beneficial effects in comparison to commonly clinically used drugs.

In conclusion, our data from behavioral assessments and LFP/EEG changes support the idea that female mice are more sensitive to neuroactive steroid-induced hypnosis. Our data also showed that neuroactive steroid-induced hypnosis has similar spectral characteristics as common general anesthetics with some unique LFP/EEG properties. While most used general anesthetics act as positive allosteric modulators of GABA_A_ receptors, they have different binding sites in comparison to neuroactive steroids and have markedly different side effects suggesting major differences in off-target binding and clinical effect [10]. Thus, we posit that future clinical use of neuroactive steroids for clinical anesthesia warrants consideration.

## Data Availability

The original contributions presented in the study are included in the article/supplementary material, further inquiries can be directed to the corresponding author.
